# Supplementary material for: The associations between adherence to the Mediterranean diet and physical fitness in young, middle-aged, and older adults: A protocol for a systematic review and meta-analysis
Source: PLoS One. 2022 Jul 8;17(7):e0271254. doi: 10.1371/journal.pone.0271254 (PMC9269745; doi:10.1371/journal.pone.0271254)
Supplement: S2 Table — Abbreviations: CRF, cardiorespiratory fitness; MD, Mediterranean Diet; MF, motor fitness; MSF, musculoskeletal fitness. (PDF) [file pone.0271254.s002.pdf]

Studies			Participants				Intervention/exposure		Outcomes		
**Reference**	**Country**	**Study Design**	**Sample size**	**Sex**	**Age**	**Adult age group**	**MD Index**	**MD foods**	**CRF**	**MSF**	**MF**
Author information and year of publication	Country	Design of the study	Number of participants for total	Number of participants for sex	Range or means ± SD (years)	Young, middle-aged, or older adults	MD scoring systems assessed	Specific MD foods analysed	Fitness component measured	Fitness component measured	Fitness component measured
