# Supplementary material for: The associations between adherence to the Mediterranean diet and physical fitness in young, middle-aged, and older adults: A protocol for a systematic review and meta-analysis
Source: PLoS One. 2022 Jul 8;17(7):e0271254. doi: 10.1371/journal.pone.0271254 (PMC9269745; doi:10.1371/journal.pone.0271254)
Supplement: Attachment — Submitted filename: Response to Reviewers.docx [file pone.0271254.s003.docx]

Maria G Grammatikopoulou

Academic Editor,

**PLOS ONE**

Enclosed you will find a revision of our manuscript: **The associations between adherence to the Mediterranean Diet and physical fitness in young, middle-aged, and older adults: a protocol for a systematic review and meta-analysis**. Manuscript Number: PONE-D-22-03601.

We would like to thank you for giving us the opportunity to revise and improve our manuscript; we also thank the reviewers for their thoughtful and constructive comments.

We have considered all of the suggestions and have incorporated them into the revised manuscript. Changes to the original manuscript are highlighted in red, and we believe our manuscript is stronger as a result of these modifications. An itemized point-by-point response to the academic editor and reviewers’ comments is presented below.

Bruno Bizzozero Peroni

Universidad de Castilla-La Mancha

Edificio Melchor Cano, Centro de Estudios Socio-Sanitarios

Santa Teresa Jornet s/n, 16071 Cuenca, Spain.

E-mail: bruno.bizzozero@uclm.es

Telephone: + (34) 969179100 ext. 4690

**Reviewer(s)' Comments to Editor:**

**Editor**

- **1.** Please ensure that your manuscript meets PLOS ONE's style requirements, including those for file naming.

**Authors**

- Thank you for the editor comment. We have reviewed and updated our citation style, changing from brackets (5) to square brackets [5]. Moreover, we have resubmitted Figure 1 based on the figures requirements by uploading our figure file to the Preflight Analysis and Conversion Engine (PACE) digital diagnostic tool.
- **2.** Please review your reference list to ensure that it is complete and correct.

**Authors**

The reference list has been reviewed as suggested and all data were complete and correct.

- **3.** We note that one or more of the authors is affiliated with the funding organization, indicating the funder may have had some role in the design, data collection, analysis or preparation of your manuscript for publication; in other words, the funder played an indirect role through the participation of the co-authors. If the funding organization did not play a role in the study design, data collection and analysis, decision to publish, or preparation of the manuscript and only provided financial support in the form of authors' salaries and/or research materials, please do the following:

a. Review your statements relating to the author contributions and ensure you have specifically and accurately indicated the role(s) that these authors had in your study. These amendments should be made in the online form.

b. Confirm in your cover letter that you agree with the following statement, and we will change the online submission form on your behalf: 
“The funder provided support in the form of salaries for authors [insert relevant initials] but did not have any additional role in the study design, data collection and analysis, decision to publish, or preparation of the manuscript. The specific roles of these authors are articulated in the ‘author contributions’ section.

**Authors**

Thank you for the editor comment. We confirmed the follow statement: *The funder provided support in the form of salaries for authors [B.B.-P., S.N.d.A.-A., M.I.L.-L.-T., and A.E.M.] but did not have any additional role in the study design, data collection and analysis, decision to publish, or preparation of the manuscript. The specific roles of these authors are stated in the ‘author contributions’ section.*

Additionally, we have reviewed and updated our statements regarding the author’s contributions and financial disclosure in the online form. Specifically, we have included the grant number of M.I.L.-L.-T.

- **3.** We note that you have stated that you will provide repository information for your data at acceptance. Should your manuscript be accepted for publication, we will hold it until you provide the relevant accession numbers or DOIs necessary to access your data. If you wish to make changes to your Data Availability statement, please describe these changes in your cover letter and we will update your Data Availability statement to reflect the information you provide.

**Authors**

We thank the editor for his/her comment. We have revised and changed the Data Availability Statement as follow: *Our article does not report data and the data availability policy is not applicable.*

**Reviewer(s)' Comments to Author:**

**Reviewer: 1**

- **1.** The methodology section must be fully rewritten and focus on important information related to results and address specifically what was exactly done to obtain the meta-analysis outcomes presented in this work. Most importantly, no results text is available in the submitted manuscript. Moreover, it is stated that “the results of this research will be submitted to a peer-reviewed journal”.

Methods: why authors used future tense? I suggest authors read back the manuscript and be consistent using past tense for descriptive information in this section.

Results: Line 202-203 No results text is available in the submitted manuscript. Moreover, it is stated that “the results of this research will be submitted to a peer-reviewed journal”

Discussion is too short and with lack of results description, it is not possible to validate arguments raised in this section.

**Authors**

We thank the reviewer for his/her comments. However, it is important to make it clear that this manuscript is a systematic review protocol. Therefore, the present objective is not to present or discuss results but to make transparent the details of the method to be used in the proposed systematic review. With this in mind, we have rigorously followed both the PRISMA-P guidelines and the instructions to authors provided by PLOS ONE.

- Line 82. “The aim of this protocol is to provide” … not sure if “protocol” suits with the work presented in this research.

**Authors**

As we have answered to the previous comment, the present manuscript is a systematic review protocol, and the terms and verb tenses used throughout the text follow the recommendations for this type of article.

- Lines 96- 112. Authors shall write a paragraph with sentence and not use bullets to state inclusion and exclusion criteria.

**Authors**

Thank you for reviewer’s comment. As suggested, we have rephrased the paragraph as follows, (Page 6):

*“To be included, studies retrieved from the peer-reviewed literature must report the following: (i) population: healthy or unhealthy adults (≥18 years); (ii) intervention/exposure: the adherence of the MD according to the overall score of different scales (e.g., Mediterranean Diet Score, Mediterranean Diet Adherence Screener) and to specific components (foods and nutrients) of these scoring systems; (iii) outcome: PF components (cardiorespiratory fitness, musculoskeletal fitness, and motor fitness) by using standardized tests; (iv) designs: observational studies (cross-sectional, case–control, prospective/retrospective cohort and longitudinal) and randomized controlled trials; and (v) period: published before January 31^st^, 2022. Moreover, studies will be excluded if they report: (i) duplicate data published in another included study; (ii) diet in terms of intake of single nutrients, food items, and food groups; (iii) special interest group data (e.g., elite athletes or firefighters); (iv) PF measured by self-report; (v) qualitative data; and (vi) data published as conference/meeting abstracts.”*

- Line 136. Figure 1 cannot be found within the manuscript or not properly labeled.

**Authors**

Thank you for reviewer’s comment. In with the submission guidelines, and specifically in relation to the figures section, we do not include Figure 1 in the main file of the manuscript but as an individual file. The legend of Figure 1 was inserted in the text of the manuscript (Page 8).

- Line 151. Scoring for adherence to Mediterranean diet shall be briefly described in addition to previous references.

**Authors**

Thank you. We have included the suggested information regarding the MD scoring systems (Page 9):

*“Both the earlier (e.g., Mediterranean Diet Score) and newer (e.g., Mediterranean Dietary Serving Score) MD scoring systems, as well as the more widely validated Mediterranean Diet Adherence Screener, will be considered for the assessment of the MD [4].”*

**Reviewer: 2**

- p.4 line 65, could you please expand?

**Authors**

The reviewer´s comment seems judicious. As suggested, we have expanded this sentence (Page 4):

*“The synergistic effect of the dietary components included in the MD scoring systems leads to a favorable nutrient intake (i.e., low contents of saturated and trans fatty acids and high contents of unsaturated fatty acids, dietary fiber, vitamins, and minerals) associated with several health benefits* ***such as better metabolic and inflammatory risk parameters****”*

- p.8 line 131: will examine

**Authors**

Thank you for the reviewer’s comment. As suggested, we have modified this sentence (Page 8).

*“Following this step and based on inclusion/exclusion criteria, two researchers* ***will*** *independently* ***examine*** *the titles and abstracts.”*

- p. 10 line 177: will be compared

**Authors**

Thank you for the reviewer’s comment. As suggested, we have modified this sentence (Page 10).

*“Once the main characteristics of the included studies have been extracted, higher vs lower MD exposures* ***will be compared*** *for each PF outcome, and these data will be synthesized narratively sub-grouped by sex, adult age group, and health status.”*

- And a suggestion: maybe you would like to present your results per age group. And maybe you could do your analysis per age group? Of course, this depends on the evaluation of different outcomes in included studies, but worth thinking about it.

**Authors**

We really appreciate the reviewer’s thoughtful comment. In fact, we consider it important to carry out, within the possibility of the data from the included studies, subgroup analyses according to the characteristics of the population such as sex, adult age group or health status (Page 10).
